# Mindfulness-based therapy improves brain functional network reconfiguration efficiency

**DOI:** 10.1038/s41398-023-02642-9

**Published:** 2023-11-11

**Authors:** Wan Lin Yue, Kwun Kei Ng, Amelia Jialing Koh, Francesca Perini, Kinjal Doshi, Juan Helen Zhou, Julian Lim

**Affiliations:** 1 Centre for Sleep and Cognition & Centre for Translational Magnetic Resonance Research, Yong Loo Lin School of Medicine, National University of Singapore, Singapore, Singapore; 2 Integrative Sciences and Engineering Programme, NUS Graduate School, National University of Singapore, Singapore, Singapore; 3 Department of Psychology, Singapore General Hospital, Singapore, Singapore; 4 Department of Electrical and Computer Engineering, National University of Singapore, Singapore, Singapore; 5 Department of Psychology, National University of, Singapore, Singapore

**Keywords:** Neuroscience, Psychology

## Abstract

Mindfulness-based interventions are showing increasing promise as a treatment for psychological disorders, with improvements in cognition and emotion regulation after intervention. Understanding the changes in functional brain activity and neural plasticity that underlie these benefits from mindfulness interventions is thus of interest in current neuroimaging research. Previous studies have found functional brain changes during resting and task states to be associated with mindfulness both cross-sectionally and longitudinally, particularly in the executive control, default mode and salience networks. However, limited research has combined information from rest and task to study mindfulness-related functional changes in the brain, particularly in the context of intervention studies with active controls. Recent work has found that the reconfiguration efficiency of brain activity patterns between rest and task states is behaviorally relevant in healthy young adults. Thus, we applied this measure to investigate how mindfulness intervention changed functional reconfiguration between rest and a breath-counting task in elderly participants with self-reported sleep difficulties. Improving on previous longitudinal designs, we compared the intervention effects of a mindfulness-based therapy to an active control (sleep hygiene) intervention. We found that mindfulness intervention improved self-reported mindfulness measures and brain functional reconfiguration efficiency in the executive control, default mode and salience networks, though the brain and behavioral changes were not associated with each other. Our findings suggest that neuroplasticity may be induced through regular mindfulness practice, thus bringing the intrinsic functional configuration in participants’ brains closer to a state required for mindful awareness.

## Introduction

Mindfulness refers to attending to what is happening in the present, while accepting and not passing judgment on these experiences [1]. Practicing mindfulness has been found to improve psychological wellbeing through beneficial effects on cognitive and emotional processes, such as improvements in working memory [2–4], attention [5–7] and emotion regulation [2, 8–10]. Furthermore, mindfulness training has been found to be effective in the treatment of psychological disorders such as depression [11–13], anxiety [12, 14–16], stress [17–19], and insomnia [20–22]. In particular, the prevalence of sleep disturbances has often been reported to increase in the elderly [for reviews, see refs. [23, 24], which is linked to increased risk of depression [25–27] and cardiovascular disease [28–31], as well as dementia and cognitive impairment [32–34]. The use of mindfulness training as a potential method for improving sleep quality may be especially relevant for elderly with sleep difficulties to reduce the risk of these disorders and improve quality of life in old age [35, 36]. In line with this, previous studies have also reported improvement in sleep quality following mindfulness-based interventions in elderly participants [22, 37–39].

To understand the mechanisms underlying these beneficial effects of mindfulness, previous studies have investigated the brain activations [for reviews, see refs. [40, 41] and functional connectivity (FC; temporal synchrony of activity across brain regions) [for reviews, see refs. [42–44] related to mindfulness. Most of these studies have focused on cross-sectional mindfulness-related functional brain differences between meditators and non-meditators or novice meditators [e.g., refs. [45–51]. Such studies reported altered activation and connectivity of default mode network regions (e.g., posterior cingulate cortex and medial prefrontal cortex), salience network regions (e.g., insula) and executive control network regions (e.g., dorsolateral prefrontal cortex) in more experienced meditators. More recent studies have adopted longitudinal designs to study functional brain changes after mindfulness-based interventions [52–59]. Changes in brain activity and functional connectivity of the default mode, salience and executive control networks were also reported in such studies, though more studies including active control interventions are needed to elucidate the specific neural correlates of mindfulness [59].

The involvement of these networks in mindfulness is consistent with the role of the default mode network in self-referential processing [60, 61], the salience network in interoceptive awareness [62, 63], and the executive control network in cognitive control and externally-directed goals [64, 65]. Furthermore, the salience network is involved in switching attentional focus between external and internal cognition [64, 66]. Since the default mode network is involved in internally oriented cognition and the executive control network is involved in externally oriented cognition, the interaction between the three networks could further support their role in switching from externally oriented attention to internally oriented mindfulness-related processes. In addition, previous studies have found that mindfulness-related measures are associated with intrinsic FC [55, 67–70] as well as task activations [71–73] in these networks. Of note, the anterior cingulate cortex (ACC) is also involved in mindfulness, and can be divided into dorsal and rostral subregions, which contribute more to the default mode network and salience network respectively. While both subregions are implicated in mindfulness conditions [74] as well as mindfulness training [43], they may mediate different mindfulness-related processes relating to their network assignments – dorsal ACC is relevant to focused attention and conscious awareness [75], while ventral ACC is involved in emotion regulation and mindwandering [43, 76].

As mindfulness is associated with functional brain activity in both rest and task, combining information from both conditions could provide further insights into the effects of mindfulness training on the brain. Indeed, recent studies have used graph theoretical methods [77–80], predictive modeling [81–83], information transfer mapping [84] and correlational approaches [85–88] to integrate rest and task fMRI data to reveal novel insights into functional brain organization supporting cognition.

Among these, a simple and straightforward way to combine rest and task information is through the measure of rest-task FC similarity (defined as the spatial Pearson correlation between rest and task FC). The rest-task FC similarity measure represents the amount of reconfiguration in brain FC patterns needed when a person goes from an intrinsic, task-free resting state to a state of performing a task [87]. Previous work has found that higher fluid intelligence and better performance across language, reasoning and working memory tasks was associated with higher rest-task FC similarity (i.e., less FC reconfiguration or more efficient FC reconfiguration from rest to task) in young adults. This suggests that individuals with better cognitive ability have intrinsic FC that is optimized for general cognition, thus requiring less functional reconfiguration from rest to task [87].

The efficiency of functional reconfiguration between rest and task could be especially relevant for studying mindfulness as mindfulness training is thought to promote increases in trait mindfulness, permitting practitioners to more easily and frequently enter a mindful state [89]. Supporting this view, previous work reported improved efficiency of FC patterns during meditation in expert compared to novice meditators [90], suggesting an easier-to-reach state of mindfulness in more practiced individuals. Furthermore, reduced activation of the default mode network regions during meditation tasks with mindfulness practice have been reported previously [46, 91]. This indicates that less activity in the default mode network is needed to support mindfulness with training, for example, due to reduced mindwandering, suggesting more efficient functional processes supporting mindfulness states in experienced meditators [46, 54, 92]. However, previous work on FC similarity focused on externally oriented tasks [85–88], and it remains unclear how functional reconfiguration efficiency may be relevant for internally oriented, mindfulness-related tasks. Furthermore, the effect of cognitive interventions on functional reconfiguration efficiency has yet to be studied.

In this work, we aimed to investigate the changes in this rest-task functional reconfiguration efficiency after a mindfulness-based intervention in a cohort of elderly participants with reported sleep difficulties. To study the intervention effects specific to mindfulness improvements, we compared the mindfulness intervention to an active control intervention (sleep hygiene education and exercise). Specifically, we investigated network-level rest-task reconfiguration efficiency between resting state and a mindfulness-related breath-counting task (BCT) across the two intervention arms. We hypothesized that increased reconfiguration efficiency would occur after mindfulness intervention, particularly in the executive control, default mode and salience networks that are often reported to be associated with externally- and internally oriented attention, interoception and mindfulness in previous literature [41, 42, 93–95]. We also sought to see whether changes in brain network reconfiguration efficiency were related to changes in mindfulness measures.

## Methods

### Participants

To investigate mindfulness-related functional brain reconfiguration, we studied healthy older adults with self-reported sleep difficulties (*N* = 127) from the Mindfulness to Improve Sleep Trial (MIST) [22]. The MIST trial was a randomized controlled study in which mindfulness-based therapy for insomnia (MBTI) was compared against an active control intervention (sleep hygiene, education and exercise program; SHEEP) for improvement of sleep quality. Sample size required to detect intervention effects were determined before recruitment based on reported effect sizes from the literature, and researchers collecting the data from the trial were blinded to the participants’ assigned intervention group [see ref. [22] for details]. The trial was approved by the SingHealth Clinical Institution Review Board and the Institutional Review Board of the National University of Singapore, as well as registered on ClinicalTrials.gov as Improving Sleep Continuity Through Mindfulness Training for Better Cognitive Ageing with the identifier NCT03677726. Written informed consent was obtained from all participants. Inclusion criteria for MIST were: (i) age within 50–80 years, (ii) English fluency, (iii) cognitively normal (Montreal Cognitive Assessment (MoCA) score [96] ≥23, Mini-Mental State Examination (MMSE) score [97] ≥26), and (iv) self-reported sleep difficulties (Pittsburgh Sleep Quality Index (PSQI) [98] score ≥5 AND >30 min sleep latency and/or >30 min wakefulness after sleep onset and/or <6.5 h sleep time). Participants were excluded if they had: (i) any neurological or psychiatric disorders, (ii) use of long-term sleep medications, (iii) prior mindfulness-based intervention, or (iv) MRI contraindications.

For this study, we chose a subset of participants from the MIST study who had resting state and breath-counting task fMRI data meeting quality control criteria before and after intervention. Specifically, 42 participants were excluded as they did not have imaging data at both pre- and post- intervention, 2 participants were excluded due to incidental findings, 1 participant withdrew from the study, and 5 participants were dropped due to incomplete data (scans were truncated or behavioral data was lost due to technical issues). 10 participants were also excluded due to poor performance during the breath-counting task inside the scanner, defined as having less than 50% accuracy for the task (see *Breath-counting task* for details). In addition, 16 participants were excluded as their imaging data did not meet the quality control criteria for head motion (1 failed for post-intervention rest scan, 11 for pre-intervention BCT scan, 4 for post-intervention BCT scan; see *Image preprocessing* for details). We further excluded 3 participants as they were outliers (>3 standard deviations from the mean for task performance or functional reconfiguration measures). This resulted in 48 participants used in our study (Table 1). We note that the subsample was representative of the full sample in the MIST study [22] based on demographics and behavioral measures – age, gender, sleep indices and Five Facet Mindfulness Questionnaire (FFMQ) scores were all not significantly different between the subsample and the full sample (two-sided Mann–Whitney U tests; all *p* > 0.05).

**Table 1:** Demographics of participants in full sample from mindfulness to improve sleep trial and subset used in this study.

Variables	MIST (full cohort)		Subset used in this study	
	MBTI	SHEEP	MBTI	SHEEP
*N*	65 (36F/29M)	62 (38F/24M)	25 (14F/11M)	23 (14F/9M)
Age (years)	61.2 ± 6.6	60.7 ± 6.2	59.9 ± 6.0	59.2 ± 5.3
Education level^a^	3.5 ± 1.1	3.5 ± 1.0	3.9 ± 0.8	3.6 ± 1.2
Ethnicity				
Chinese	63	57	24	19
Malay	0	0	0	0
Indian	1	1	0	0
Other	1	4	1	4
PSQI				
Pre	10.98 ± 3.10^b^	10.87 ± 3.10^b^	10.36 ± 3.26^b^	10.91 ± 3.03
Post	7.34 ± 3.01^b^	7.58 ± 3.37^b^	6.48 ± 2.93^b^	8.09 ± 3.69^c^
ISI				
Pre	14.89 ± 3.89^b^	14.21 ± 4.13^b^	15.20 ± 3.24^b^	14.30 ± 3.18^b^
Post	9.95 ± 3.88^b^	11.23 ± 4.54^b^	9.20 ± 4.20^b^	12.52 ± 4.14^b,c^
FFMQ				
Pre	128.83 ± 17.13	131.68 ± 16.38	125.48 ± 15.28	128.87 ± 11.17
Post	131.43 ± 16.59	132.61 ± 14.30	131.92 ± 14.23	132.52 ± 13.42

All values are listed as mean ± standard deviation (except number of participants). PSQI and ISI are primary outcomes in the Mindfulness to Improve Sleep Trial (MIST), while FFMQ is a secondary outcome.

*MBTI* Mindfulness-Based Therapy for Insomnia, *SHEEP* Sleep Hygiene Exercise and Education Program, *PSQI* Pittsburgh Sleep Quality Index, *ISI* Insomnia Symptoms Index, *FFMQ* Five-Facets Mindfulness Questionnaire.

^a^Education was categorized into 6 levels as follows: 0 = no education, 1 = less than or equals primary education, 2 = less than or equals secondary education, 3 = more than secondary or less than university education, 4 = university-level education, 5 = post graduate-level education.

^b^Significant differences (*p* < 0.05) pre- and post-intervention from Mann–Whitney U test.

^c^Significant differences (*p* < 0.05) across MBTI and SHEEP intervention groups from Mann–Whitney U test.

### Interventions

To compare the effect of mindfulness against an active control, participants were randomized into one of two intervention groups using a simple randomization procedure in MATLAB. Both interventions were run in weekly 2-hour sessions over 8 weeks, and matched as closely as possible for contact time, daily practice, and sleep education content. The mindfulness-based intervention was based on the MBTI developed by Ong [99], and conducted by a trained and certified mindfulness instructor. The mindfulness practices included mindful eating, mindful movement and meditation. The active control SHEEP intervention was developed at the Singapore General Hospital and conducted by a clinical psychologist. This intervention involved adaptations of habits and environment to improve sleep, and included sleep-promoting exercises (stretching, breathing exercises and muscle relaxation). More details on the interventions and randomization procedure are reported in Perini, Wong [22]. We note that there are no significant differences for sleep indices (PSQI or ISI) or FFMQ scores between the two intervention groups at baseline (two-sided Mann–Whitney U tests; all *p* > 0.05).

### Five facets mindfulness questionnaire

To obtain behavioral scores for mindfulness, self-reported measures were collected as a secondary outcome in the MIST trial using the FFMQ [100]. In brief, the FFMQ examines five facets or components of mindfulness, namely ‘Observing’, ‘Describing’, ‘Acting with awareness’, ‘Nonjudging of inner experiences’ and ‘Nonreactivity to inner experience’ (each with 8 items, except non-reactivity with 7 items). Participants rated each item on a scale of 1–5 (from ‘Never or very rarely true’ to ‘Very often or always true’). Subscale scores were obtained by taking the sum of the rating for all items within each facet, and total FFMQ scores were calculated as the sum of all subscale scores for each participant.

### Breath-counting task

To obtain functional brain measures of mindfulness, participants underwent fMRI scanning while performing an internally oriented, mindfulness-related BCT task [101, 102]. In this task, participants attended to the sensations of their breathing while using a part of their attention to maintain a count in cycles. They pressed a button to indicate breath counts 1–8 in each cycle, and a second button to indicate the 9^th^ breath. A third button was pressed if they lost count. Participants were first given instructions on the task and performed it outside of the scanner to ensure they understood the instructions. During the BCT scan, participants were instructed to keep their eyes open and perform the same task. A blank screen was shown throughout the BCT scan. BCT accuracy inside and outside of the scanner were used as task performance measures. A correct cycle comprised exactly eight presses on the first button, followed by one press on a second button. Accuracy was calculated as the number of correct cycles divided by the total number of cycles. Full details of the scoring procedure are reported in refs. [103, 104].

### Image acquisition

All participants were scanned using a 3T Siemens Magnetom Prisma Fit scanner. High-resolution T1 structural magnetic resonance imaging (MRI) scans were collected using a magnetization-prepared rapid gradient echo sequence (TR/TE = 2300/2.28 ms, voxel size = 1.0 × 1.0 × 1.0 mm^3^, FOV = 256 × 240 mm^2^, 192 sagittal slices, flip angle = 8^◦^, bandwidth = 200 Hz/pixel). Resting state and BCT task fMRI data were collected during scans of 10 min each using the same interleaved multiband echo-planar imaging sequence (TR/TE = 719/30 ms, multiband acceleration factor = 4, voxel size = 3.0 × 3.0 × 3.0 mm^3^, FOV = 225 × 225 mm^2^, 44 axial slices, flip angle = 52^◦^, bandwidth = 2632 Hz/pixel). During resting state, participants fixated on a cross presented in the center of the screen and were instructed that they need not think about anything in particular.

### Image preprocessing

Preprocessing of fMRI scans was performed using FreeSurfer [105], FSL [106, 107], and SPM (Wellcome Department of Cognitive Neurology, London, UK) through the pipeline developed by the Computational Brain Imaging Group [108]. In brief, the functional images underwent removal of four volumes from the start of each run; motion correction; boundary-based registration onto Freesurfer surface; creation of whole brain, white matter and ventricular masks from structural segmentation; regression of nuisance signals (whole brain, white matter signal, ventricle signal and 12 motion parameters); band pass filtering (0.009–0.08 Hz); projection onto MNI-152 volumetric space; downsampling to 2 mm voxels; and spatial smoothing with a Gaussian kernel (6 mm full-width half maximum). For consistency, the same preprocessing steps were performed for both rest and BCT task fMRI scans.

Functional images were subjected to quality control for head motion using measures of framewise displacement (FD) and variance of temporal derivative of time courses over voxels (DVARS) [109]. Volumes with high motion (FD > 0.2 mm or DVARS > 50 [110, 111]), one frame before and two frames after the high motion volumes were interpolated from surrounding data. Participants were excluded if <50% of the original frames (i.e., <5 min of each scan [112]) in the full run remained after this motion correction step. The final sample had mean relative rest motion = 0.07 ± 0.02 mm and mean relative task motion = 0.10 ± 0.03 mm. No significant group, time or interaction effects were observed in mean relative rest and task motion.

### Derivation of functional reconfiguration

Following previous work on rest-task functional reconfiguration efficiency [87, 88], we further regressed task activations from preprocessed task fMRI data before deriving task FC [113]. Specifically, main and derivative regressors were created for button presses recorded during the BCT, which were then convolved with a canonical hemodynamic response function.

To extract fMRI time series for construction of FC matrices, we used a parcellation with 400 cortical regions of interest (ROIs) [114] and 30 subcortical ROIs [115, 116], averaging over voxels within each ROI to obtain ROI-level time series. The ROIs were then grouped into 9 networks (executive control, default mode, dorsal attention, limbic, salience/ventral attention, somatomotor, temporal-parietal, visual and subcortical). We calculated Pearson’s correlation for each pair of ROI time series (Fisher’s r-to-z transformed) to represent FC between ROIs. We then constructed rest and BCT FC matrices for each participant using the correlation z-values from resting state and BCT respectively.

We studied rest-task functional reconfiguration efficiency through network-level FC similarity measures. Network-level FC similarity was separated into intranetwork and internetwork components. Specifically, for intranetwork, rest-task FC similarity was computed as the Pearson’s correlation (Fisher’s r-to-z transformed) of all FC between ROIs of each network in the resting state with all FC between ROIs of the same network in the task state. For internetwork, rest-task FC similarity was computed as the Pearson’s correlation (Fisher’s r-to-z transformed) of the between-network FC vector (i.e., all the FC values between ROIs from one network and ROIs of all other networks) in the resting state with the same between-network FC vector in the task state. For each network and each participant at both pre- and post-intervention sessions, this yielded one intranetwork and one internetwork FC similarity value. Higher rest-task FC similarity corresponds to less reconfiguration of spatial patterns of brain between rest and task, or more efficient functional reconfiguration.

### Statistical analyses

To investigate behavioral and neuroimaging measures before and after the two interventions, we performed linear mixed modeling separately on BCT performance (accuracy inside and outside of scanner), FFMQ scores (subscale scores and total score) and FC similarity measures (network-level):

$$Y \sim {Age}+{Gender}+{Group}* {Timepoint}+\left(1+{Timepoint|Participant}\right)$$

Where Y represented the outcome measure of interest, Age was the participants’ age at the start of the intervention, while Gender, Group and Timepoint were binary dummy variables for the participants’ gender, intervention group, and pre- or post-intervention timepoints. Data from all participants were included in each model. The models also included random intercepts and slopes for each participant. The coefficient (*β*) for Timepoint was the estimated time effect for changes in the outcome before and after the interventions. Importantly, the interaction term (Group x Timepoint, as enclosed in the * notation) was included to assess if the changes were different between the two intervention groups.

Next, we investigated whether any behavioral changes were positively associated with FC similarity changes across all participants. This was performed only for behavioral and neuroimaging measures that showed a time and/or interaction effect in the previous analysis. Linear models were constructed with behavioral change as the outcome variable and FC similarity change as the independent variable. Age and sex were included as covariates of no interest. To assess if there were differential associations between brain and behavior across the two intervention groups, a second set of models were also constructed with group interacting with FC similarity change.

Due to the relatively small sample size, we used non-parametric methods to obtain *p* values for the described analyses. Specifically, we performed permutation analyses to obtain null distributions of estimates, shuffling Group labels across all participants and switching Timepoint labels within participants 50% of the time for 1000 iterations. The *p* values were then calculated as the fraction of iterations in which the permuted estimates were as extreme as or more extreme than the observed estimate. All statistical analyses described were carried out in MATLAB 2015a (The MathWorks, Inc.), and the code is available on Github (https://github.com/hzlab/2022_Yue_Mindfulness_Reconfiguration_TransPsy). Data used in this study is available upon reasonable request from the corresponding authors.

## Results

### Effects of interventions on mindfulness measures

**Fig. 1:**
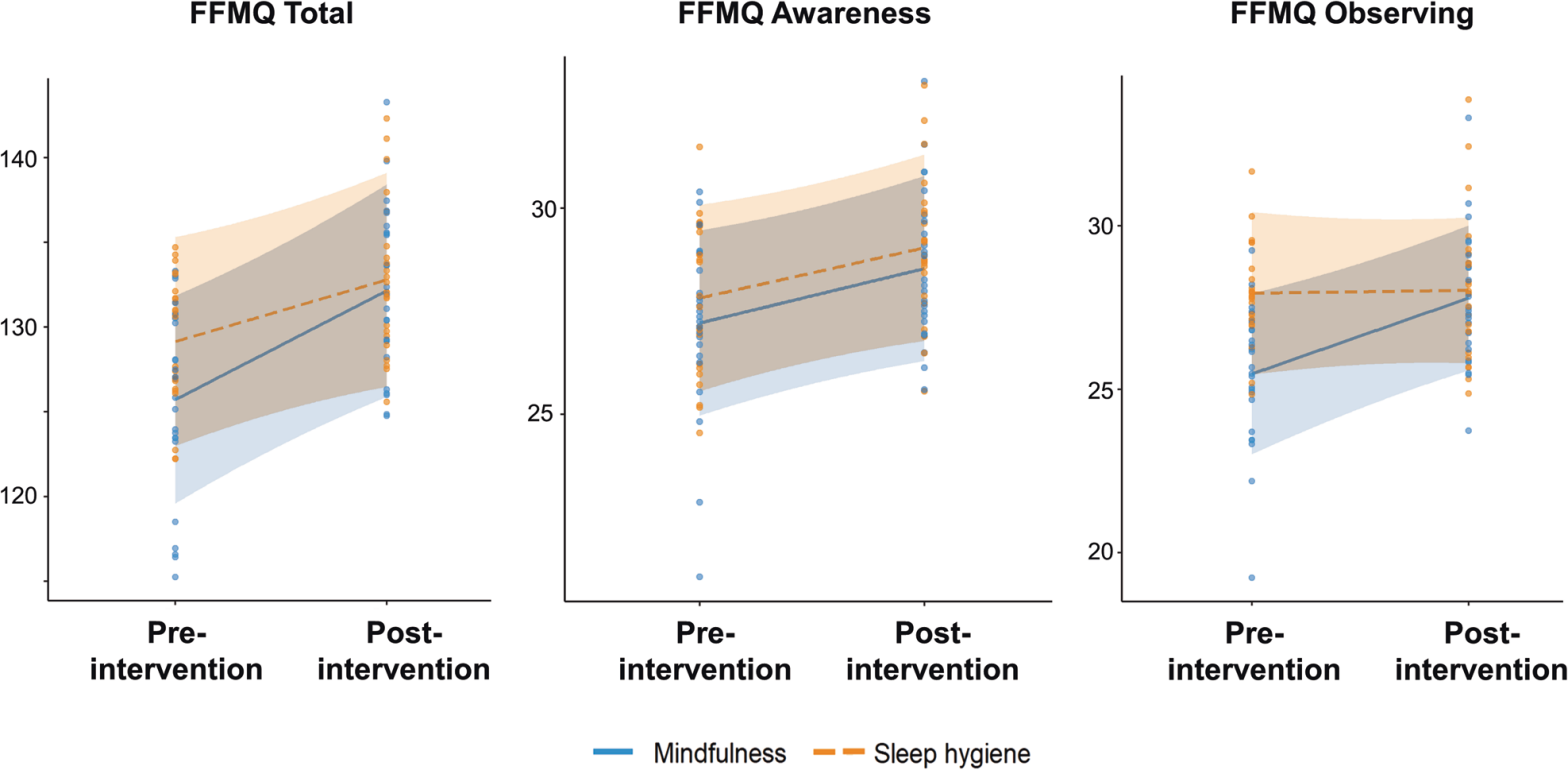
Self-reported mindfulness measures improved after intervention. FFMQ Total score and FFMQ Awareness subscale score showed Time effect and increased after intervention for both groups (FFMQ Total: *β*_Timepoint_ = 6.44, Cliff’s δ_Post-Pre_ effect size = 0.19, *p* < 0.001, FFMQ Awareness: *β*_Timepoint_ = 2.32, δ_Post-Pre_ = 0.11, *p* = 0.001). FFMQ Observing subscale score showed an interaction effect, with an increase in subscale score only in the mindfulness intervention group but not the sleep hygiene group (*β*_Group × Timepoint_ = −2.23, δ_MBTI(Post- Pre)_ = 0.30, δ_SHEEP(Post- Pre)_ = −0.004, *p* = 0.042). Only significant effects from linear mixed models (*p* < 0.05 from 1000 permutations, controlled for age and gender) are illustrated, and residuals from the linear mixed model (controlling for age and gender) are plotted here. All time and interaction effects for behavior are detailed in Supplementary Table 1.

Overall, we found that both the FFMQ total score and the FFMQ Awareness and Observing subscale scores increased after intervention across all participants (Fig. 1, FFMQ Total: *β*_Timepoint_ = 6.44, Cliff’s δ_Post-Pre_ effect size = 0.19, *p* < 0.001, FFMQ Awareness: *β*_Timepoint_ = 1.32, δ_Post-Pre_ = 0.11, *p* = 0.044, FFMQ Observing: *β*_Timepoint_ = 2.32, δ_Post-Pre_ = 0.14, *p* = 0.001). For the Observing subscale, we further found an interaction effect such that the subscale score appeared to increase only in the mindfulness intervention group but not the sleep hygiene group (*β*_Group × Timepoint_ = −2.23, δ_MBTI(Post- Pre)_ = 0.30, δ_SHEEP(Post- Pre)_ = −0.004, *p* = 0.032), suggesting that the time effect for FFMQ Observing subscale was driven by the improvement in the mindfulness intervention group. All other mindfulness measures (from FFMQ and BCT) did not show significant time and/or interaction effects, while sleep measures of PSQI showed a time effect and Insomnia Severity Index (ISI) [117] showed both time and intervention effects (Supplementary Table 1).

### Effects of interventions on functional reconfiguration efficiency

**Fig. 2:**
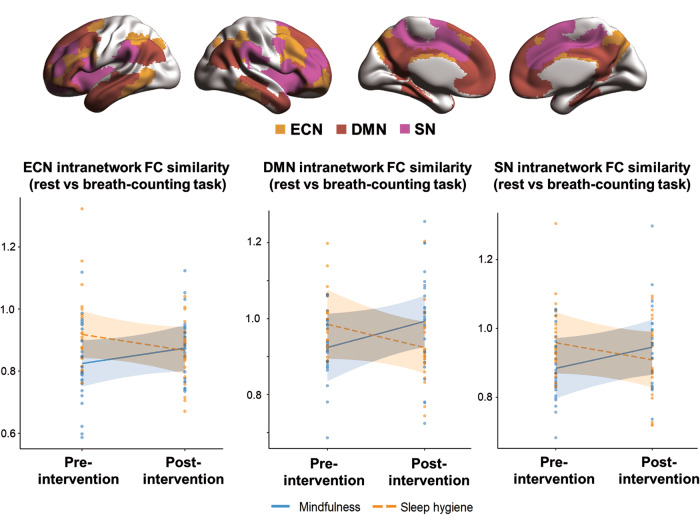
Brain functional reconfiguration efficiency improved after mindfulness intervention. Interaction effects were observed for intranetwork FC similarity between rest and breath-counting task (BCT) in brain networks (executive control network (ECN): *β*_Group × Timepoint_ = −0.10, Cliff’s δ_MBTI(Post-Pre)_ effect size = 0.16, δ_SHEEP(Post-Pre)_ = −0.17, *p* = 0.032, default mode network (DMN): *β*_Group × Timepoint_ = −0.13, δ_MBTI(Post-Pre)_ = 0.21, δ_SHEEP(Post-Pre)_ = −0.10, *p* = 0.013, salience network (SN): *β*_Group × Timepoint_ = −0.11, δ_MBTI(Post-Pre)_ = 0.16, δ_SHEEP(Post-Pre)_ = −0.10, *p* = 0.027). Both networks increased in FC similarity in the mindfulness group but decreased in the sleep hygiene group. Only significant interaction effects from linear mixed models (*p* < 0.05 from 1000 permutations, controlled for age and gender) are illustrated; all time and interaction effects for FC similarity are detailed in Supplementary Table 2. FC similarity was calculated using data preprocessed with global signal and task activations regressed, and residuals from the linear mixed model (controlling for age and gender) are plotted here. Brain networks were visualized using BrainNet Viewer (http://www.nitrc.org/projects/bnv).

Brain FC similarity between rest and BCT task showed differential changes after intervention for the mindfulness and sleep hygiene groups. Specifically, we observed group and time interaction effects for rest-task intranetwork FC similarity in the executive control network (*β*_Group × Timepoint_ = −0.10, δ_MBTI(Post- Pre)_ = 0.16, δ_SHEEP(Post- Pre)_ = −0.17, *p* = 0.032), default mode network (*β*_Group × Timepoint_ = −0.13, δ_MBTI(Post- Pre)_ = 0.21, δ_SHEEP(Post- Pre)_ = −0.10, *p* = 0.013) and salience network (*β*_Group × Timepoint_ = −0.11, δ_MBTI(Post- Pre)_ = 0.16, δ_SHEEP(Post- Pre)_ = −0.10, *p* = 0.027), such that FC similarity appeared to increase over time for mindfulness intervention group but seemed to decrease in the sleep hygiene group within all three networks (Fig. 2).

Since the intranetwork FC similarity of these networks were not significantly different across the two groups pre-intervention (two-tailed Mann–Whitney U test; all *p* > 0.05), baseline differences did not contribute to the observed interaction effects. FC similarity measures from all other networks (both intranetwork and internetwork) did not show significant time and/or interaction effects (Supplementary Table 2). Furthermore, repeating the analyses with network-averaged rest and task FC revealed interaction effects only in the default mode network for rest FC and the somatomotor network for task FC (Supplementary Table 3), indicating that changes in FC similarity between rest and task were not simply due to changes in rest or task FC alone.

### Associations between changes in mindfulness measures and reconfiguration efficiency

Finally, we found no positive associations between these behavioral and brain imaging measures, and no interaction effects on associations across intervention groups (all *p* > 0.05 from 1000 permutations; Supplementary Table 4). Similarly, no associations were found with sleep measures (Supplementary Table 5).

## Discussion

In this study, we investigated how mindfulness intervention changed network-level functional reconfiguration between resting state and a breath-counting task in a sample of healthy older adults with reported sleep difficulties. Overall, we found that self-reported mindfulness measures and brain functional reconfiguration efficiency in the executive control, default mode and salience networks improved after mindfulness intervention, though the behavioral and brain changes did not show significant associations with each other. Our findings suggest that regular mindfulness practice may induce changes in neuroplasticity of the executive control, default mode and salience networks, such that the participants’ intrinsic functional brain configurations were brought closer to a state of mindful awareness.

Our findings add to the existing evidence that mindfulness improves after short-term practice, with self-reported mindfulness measures proving useful for assessing effects of mindfulness-based intervention [118]. Specifically, we found that FFMQ Observing subscale score improved only for the mindfulness group and not the control group, indicating that formal mindfulness practice did contribute to improvements in mindfulness over the control intervention. This result mirrors the group × time interaction found for FFMQ in the full MIST sample, which was driven by increased FFMQ scores in the mindfulness group [22].

Our findings of higher rest-task functional network reconfiguration efficiency after mindfulness intervention suggest that mindfulness practice made it easier (or less effortful) for participants in the mindfulness group to move from resting state to a mindful state for performing the BCT, even if there was no significant difference in their BCT performance. The observed increase in functional reconfiguration efficiency after mindfulness intervention is consistent with the previously reported results on functional reconfiguration with externally oriented tasks, where participants with more efficient rest-task functional reconfiguration had better task performance and general cognitive abilities [87, 88]. In addition, rest-task functional reconfiguration efficiency appeared to increase after the mindfulness intervention but not the active control intervention. This may indicate that participants who underwent mindfulness practice and sleep hygiene education had differential functional brain reorganization based on the type of training received. Since the sleep hygiene intervention did not include mindfulness techniques, different strategies could have been used that caused their rest and BCT task states to differ more after the intervention. Furthermore, previous work using graph-theoretical methods to study FC during meditation has reported that brain regions show more efficient FC organization in expert compared to novice meditators [90], providing further support for our findings of improved functional brain reorganization from rest to task in participants who underwent mindfulness intervention.

Our observations of intervention effects in the executive control, default mode and salience networks are consistent with existing literature on the relevance of these networks to mindfulness. The default mode network is involved in self-referential processes and mind-wandering [60, 61]. Regions in the default mode network have been observed to have reduced activity during mindfulness tasks [46, 48, 51, 72], with further reductions in activity in experienced meditators [46] possibly reflecting reduced mindwandering, thus suggesting increased efficiency in this network with mindfulness practice. On the other hand, the key regions of the salience network – anterior insula and anterior cingulate cortex – have roles in interoception and self-awareness [62, 63], which is especially relevant to the BCT used in this study. In previous work, the regions of the salience network have also been found to be consistently activated during meditation [for review, see ref. [41]. The executive control network is involved in cognitive control and externally-directed goals [64, 65], and has also been linked to mindfulness in previous literature [49, 58, 67, 71]. Further supporting the involvement of the executive control, default mode and salience networks in mindfulness practice, mindfulness-related changes were also observed for FC within and between these networks during mindfulness tasks as well as resting state in previous studies [46, 47, 52, 119, 120]. In particular, mindfulness-related increases in FC within these networks in resting state [for reviews, see refs. [43, 46] suggest that our observed intervention effects on rest-task functional reconfiguration efficiency can be explained by the intrinsic functional organization of these networks getting closer to a mindful state.

In the context of mindfulness-based tasks like the BCT, functional reconfiguration in the default mode network from resting state to task could be related to transitions towards a more self-detached viewpoint as well as increasing focus through reduced mind-wandering, which become easier to achieve after mindfulness intervention. Crucially, the salience network is involved in switching between internally- and externally oriented attention [64, 66], which is relevant for attentional processes during mindfulness states that also become more efficient with mindfulness practice [54, 92]. Since the default mode network is involved in internal cognition while the executive control network is involved in externally-directed cognition, the three networks could be working together as the participants switch from a state of attending to stimuli from the environment into a self-aware state during mindfulness tasks. Taken together, changes in network-level FC from rest to task may thus correspond to the involvement of the three networks in key processes related to mindfulness practice such as increased self-awareness, decentering and focused attention. Improvements in the executive control network, default mode network and salience network FC reconfiguration efficiency from pre- to post-intervention could thus be a potential mechanism underlying observed improvements in mindfulness measures.

Despite the observed changes in both mindfulness measures and functional reconfiguration efficiency post-intervention, we did not find any significant associations between these changes. Similarly, a previous study found cross-sectional differences in task performance and neural responses between meditators and non-meditators, but no significant correlations between these differences [121]. The lack of brain-behavior associations may be due to behavioral and neuroimaging measures changing at different timescales [122]. Future studies could include additional follow-up sessions which may reveal associations between these measures at different timepoints post-intervention and whether functional brain changes were maintained.

### Limitations and future directions

Our study had a moderate sample size which may not be sufficiently powered to detect the associations between changes in brain and behavioral measures before and after intervention. Larger sample sizes or meta-analyses may be required to reveal these associations. Furthermore, as only older adults were studied, future work could investigate possible differences in mindfulness-related functional reconfiguration in aging and development.

Moreover, we note that the FFMQ total score increased in both intervention groups instead of showing improvements only for the mindfulness intervention group. This may be due to the poor discriminant validity of the FFMQ measure, which has been also reported in previous literature [123, 124]. Furthermore, the FFMQ suffers from several limitations due to its nature as a self-report measure. For example, self-report measures of mindfulness are confounded by differing interpretations of questionnaire items across participants as well as before and after mindfulness practice [125–127]. Participants may also misrepresent themselves on the questionnaire, either intentionally or unintentionally. In addition, filling up the questionnaire may require a certain level of mindfulness, and may also positively affect one’s mindfulness levels [128]. Despite these inherent limitations, the FFMQ is still considered the gold standard among mindfulness measures and is widely used in the field. Thus, future work on developing better measures for assessing mindfulness is needed to advance our understanding of the specific neural mechanisms underlying the beneficial effects of mindfulness.

We further note that the use of FC to study the neural correlates of mindfulness also poses several limitations. First, the network-level or region-specific changes observed may be task-specific due to different cognitive processes being involved to different extents across mindfulness tasks [129]. Future work could investigate functional reconfiguration efficiency across a variety of mindfulness-based tasks to obtain a more complete picture of how network-level functional reconfiguration supports mindfulness. Second, neuroimaging confounds such as head motion and physiological changes could affect the estimation of FC and FC-derived measures, with mindfulness possibly also changing the impact of these confounds on the neuroimaging measures [130]. While we selected only participants with minimal head motion for inclusion in this study, physiological measures such as heart rate could also be taken into account for future work, especially given the interoceptive nature of mindfulness-based tasks. Third, we only used static FC to study mindfulness-related changes in functional reconfiguration, but previous work revealed that FC patterns do not remain constant throughout mindfulness states and their dynamics are relevant to mindfulness measures [131–133]. Future work could thus investigate how the dynamics of functional reconfiguration may also be relevant for supporting mindfulness-related processes.

In conclusion, our study revealed improvements in self-reported mindfulness scores and increased rest-task functional brain reconfiguration efficiency of the executive control, default mode and salience networks through mindfulness practice. These findings were specific to mindfulness training as the active control intervention group did not show these changes. Our findings suggest that participants’ brain functional organization becomes more efficient and closer to a state of mindful awareness through undergoing mindfulness intervention, and indicate that neuroplasticity may be induced through mindfulness practice.

### Supplementary information

Supplementary materials

## References

[1] Kabat-Zinn J. Full catastrophe living: using the wisdom of your body and mind to face stress, pain, and illness, New York, N.Y.: Delacorte Press; 1990.

[2] Jha AP, Stanley EA, Kiyonaga A, Wong L, Gelfand L (2010). Examining the protective effects of mindfulness training on working memory capacity and affective experience. Emotion.

[3] Jha AP, Witkin JE, Morrison AB, Rostrup N, Stanley E (2017). Short-form mindfulness training protects against working memory degradation over high-demand intervals. J Cogn Enhancement.

[4] Mrazek MD, Franklin MS, Phillips DT, Baird B, Schooler JW (2013). Mindfulness training improves working memory capacity and GRE performance while reducing mind wandering. Psychological Sci.

[5] Jha AP, Krompinger J, Baime MJ (2007). Mindfulness training modifies subsystems of attention. Cogn Affect Behav Neurosci.

[6] Moore A, Malinowski P (2009). Meditation, mindfulness and cognitive flexibility. Conscious Cogn.

[7] Zeidan F, Johnson SK, Diamond BJ, David Z, Goolkasian P (2010). Mindfulness meditation improves cognition: evidence of brief mental training. Conscious Cogn.

[8] Arch JJ, Craske MG (2006). Mechanisms of mindfulness: emotion regulation following a focused breathing induction. Behav Res Ther.

[9] Brown KW, Ryan RM (2003). The benefits of being present: mindfulness and its role in psychological well-being. J Personal Soc Psychol.

[10] Hill CL, Updegraff JA (2012). Mindfulness and its relationship to emotional regulation. Emotion.

[11] Barnhofer T, Crane C, Hargus E, Amarasinghe M, Winder R, Williams JMG (2009). Mindfulness-based cognitive therapy as a treatment for chronic depression: a preliminary study. Behav Res Ther.

[12] Hofmann SG, Sawyer AT, Witt AA, Oh D (2010). The effect of mindfulness-based therapy on anxiety and depression: a meta-analytic review. J Consult Clin Psychol.

[13] Kenny MA, Williams JMG (2007). Treatment-resistant depressed patients show a good response to mindfulness-based cognitive therapy. Behav Res Ther.

[14] Boettcher J, Astrom V, Pahlsson D, Schenstrom O, Andersson G, Carlbring P (2014). Internet-based mindfulness treatment for anxiety disorders: a randomized controlled trial. Behav Ther.

[15] Miller JJ, Fletcher K, Kabat-Zinn J (1995). Three-year follow-up and clinical implications of a mindfulness meditation-based stress reduction intervention in the treatment of anxiety disorders. Gen Hosp Psychiatry.

[16] Vøllestad J, Sivertsen B, Nielsen GH (2011). Mindfulness-based stress reduction for patients with anxiety disorders: evaluation in a randomized controlled trial. Behav Res Ther.

[17] Praissman S (2008). Mindfulness-based stress reduction: a literature review and clinician’s guide. J Am Acad Nurse Pr.

[18] Shapiro SL, Brown KW, Thoresen C, Plante TG (2011). The moderation of mindfulness-based stress reduction effects by trait mindfulness: results from a randomized controlled trial. J Clin Psychol.

[19] Walach H, Nord E, Zier C, Dietz-Waschkowski B, Kersig S, Schüpbach H (2007). Mindfulness-based stress reduction as a method for personnel development: a pilot evaluation. Int J Stress Manag.

[20] Gong H, Ni CX, Liu YZ, Zhang Y, Su WJ, Lian YJ (2016). Mindfulness meditation for insomnia: a meta-analysis of randomized controlled trials. J Psychosom Res.

[21] Ong JC, Manber R, Segal Z, Xia Y, Shapiro S, Wyatt JK (2014). A randomized controlled trial of mindfulness meditation for chronic insomnia. Sleep.

[22] Perini F, Wong KF, Lin J, Hassirim Z, Ong JL, Lo J (2023). Mindfulness-based therapy for insomnia for older adults with sleep difficulties: a randomized clinical trial. Psychol Med.

[23] Gulia KK, Kumar VM (2018). Sleep disorders in the elderly: a growing challenge. Psychogeriatrics.

[24] Suzuki K, Miyamoto M, Hirata K (2017). Sleep disorders in the elderly: diagnosis and management. J Gen Fam Med.

[25] Becker NB, Jesus SN, Joao K, Viseu JN, Martins RIS (2017). Depression and sleep quality in older adults: a meta-analysis. Psychol Health Med.

[26] Smagula SF, Reynolds CF, Ancoli-Israel S, Barrett-Connor E, Dam TT, Hughes-Austin JM (2015). Sleep architecture and mental health among community-dwelling older men. J Gerontol B Psychol Sci Soc Sci.

[27] Yu DS (2010). Insomnia Severity Index: psychometric properties with Chinese community-dwelling older people. J Adv Nurs.

[28] Huang T, Mariani S, Redline S (2020). Sleep irregularity and risk of cardiovascular events: the multi-ethnic study of atherosclerosis. J Am Coll Cardiol.

[29] Ikehara S, Iso H, Date C, Kikuchi S, Watanabe Y, Wada Y (2009). Association of sleep duration with mortality from cardiovascular disease and other causes for Japanese men and women: the JACC study. Sleep.

[30] Sabanayagam C, Shankar A, Buchwald D, Goins RT (2011). Insomnia symptoms and cardiovascular disease among older American Indians: the Native Elder Care Study. J Environ Public Health.

[31] Suzuki E, Yorifuji T, Ueshima K, Takao S, Sugiyama M, Ohta T (2009). Sleep duration, sleep quality and cardiovascular disease mortality among the elderly: a population-based cohort study. Prev Med.

[32] Dzierzewski JM, Dautovich N, Ravyts S (2018). Sleep and cognition in older adults. Sleep Med Clin.

[33] Spira AP, Chen-Edinboro LP, Wu MN, Yaffe K (2014). Impact of sleep on the risk of cognitive decline and dementia. Curr Opin Psychiatry.

[34] Yaffe K, Falvey CM, Hoang T (2014). Connections between sleep and cognition in older adults. Lancet Neurol.

[35] Driscoll HC, Serody L, Patrick S, Maurer J, Bensasi S, Houck PR (2008). Sleeping well, aging well: a descriptive and cross-sectional study of sleep in “successful agers” 75 and older. Am J Geriatr Psychiatry.

[36] Gothe NP, Ehlers DK, Salerno EA, Fanning J, Kramer AF, McAuley E (2020). Physical activity, sleep and quality of life in older adults: influence of physical, mental and social well-being. Behav Sleep Med.

[37] Black DS, O’Reilly GA, Olmstead R, Breen EC, Irwin MR (2015). Mindfulness meditation and improvement in sleep quality and daytime impairment among older adults with sleep disturbances: a randomized clinical trial. JAMA Intern Med.

[38] Camino M, Satorres E, Delhom I, Real E, Abella M, Melendez JC (2022). Mindfulness-based cognitive therapy to improve sleep quality in older adults with insomnia. Psychosoc Intervention.

[39] Li X, Liu P, Hong F (2020). A randomized controlled trial of group mindfulness therapy on sleep quality of the elderly in nursing homes. Life Res.

[40] Boccia M, Piccardi L, Guariglia P (2015). The meditative mind: a comprehensive meta-analysis of MRI studies. Biomed Res Int.

[41] Fox KC, Dixon ML, Nijeboer S, Girn M, Floman JL, Lifshitz M (2016). Functional neuroanatomy of meditation: a review and meta-analysis of 78 functional neuroimaging investigations. Neurosci Biobehav Rev.

[42] Marchand WR (2014). Neural mechanisms of mindfulness and meditation: evidence from neuroimaging studies. World J Radio.

[43] Sezer I, Pizzagalli DA, Sacchet MD (2022). Resting-state fMRI functional connectivity and mindfulness in clinical and non-clinical contexts: a review and synthesis. Neurosci Biobehav Rev.

[44] Zsadanyi SE, Kurth F, Luders E (2021). The effects of mindfulness and meditation on the cingulate cortex in the healthy human. Brain Rev Mindfulness.

[45] Brefczynski-Lewis JA, Lutz A, Schaefer HS, Levinson DB, Davidson RJ (2007). Neural correlates of attentional expertise in long-term meditation practitioners. Proc Natl Acad Sci USA.

[46] Brewer JA, Worhunsky PD, Gray JR, Tang YY, Weber J, Kober H (2011). Meditation experience is associated with differences in default mode network activity and connectivity. Proc Natl Acad Sci USA.

[47] Farb NA, Segal ZV, Anderson AK (2013). Mindfulness meditation training alters cortical representations of interoceptive attention. Soc Cogn Affect Neurosci.

[48] Garrison KA, Zeffiro TA, Scheinost D, Constable RT, Brewer JA (2015). Meditation leads to reduced default mode network activity beyond an active task. Cogn Affect Behav Neurosci.

[49] Hasenkamp W, Barsalou LW (2012). Effects of meditation experience on functional connectivity of distributed brain networks. Front Hum Neurosci.

[50] Taylor VA, Daneault V, Grant J, Scavone G, Breton E, Roffe-Vidal S (2013). Impact of meditation training on the default mode network during a restful state. Soc Cogn Affect Neurosci.

[51] Taylor VA, Grant J, Daneault V, Scavone G, Breton E, Roffe-Vidal S (2011). Impact of mindfulness on the neural responses to emotional pictures in experienced and beginner meditators. Neuroimage.

[52] Kilpatrick LA, Suyenobu BY, Smith SR, Bueller JA, Goodman T, Creswell JD (2011). Impact of Mindfulness-Based Stress Reduction training on intrinsic brain connectivity. Neuroimage.

[53] Kirlic N, Cohen ZP, Tsuchiyagaito A, Misaki M, McDermott TJ, Aupperle RL (2022). Self-regulation of the posterior cingulate cortex with real-time fMRI neurofeedback augmented mindfulness training in healthy adolescents: a nonrandomized feasibility study. Cogn Affect Behav Neurosci.

[54] Kozasa EH, Balardin JB, Sato JR, Chaim KT, Lacerda SS, Radvany J (2018). Effects of a 7-day meditation retreat on the brain function of meditators and non-meditators during an attention task. Front Hum Neurosci.

[55] Kral TRA, Imhoff-Smith T, Dean DC, Grupe D, Adluru N, Patsenko E (2019). Mindfulness-based stress reduction-related changes in posterior cingulate resting brain connectivity. Soc Cogn Affect Neurosci.

[56] Kwak S, Kim SY, Bae D, Hwang WJ, Cho KIK, Lim KO (2019). Enhanced attentional network by short-term intensive meditation. Front Psychol.

[57] Sevinc G, Holzel BK, Hashmi J, Greenberg J, McCallister A, Treadway M (2018). Common and dissociable neural activity after mindfulness-based stress reduction and relaxation response programs. Psychosom Med.

[58] Tomasino B, Fabbro F (2016). Increases in the right dorsolateral prefrontal cortex and decreases the rostral prefrontal cortex activation after-8 weeks of focused attention based mindfulness meditation. Brain Cogn.

[59] Allen M, Dietz M, Blair KS, van Beek M, Rees G, Vestergaard-Poulsen P (2012). Cognitive-affective neural plasticity following active-controlled mindfulness intervention. J Neurosci.

[60] Buckner RL, Andrews-Hanna JR, Schacter DL (2008). The brain’s default network: anatomy, function, and relevance to disease. Ann NY Acad Sci.

[61] Raichle ME, MacLeod AM, Snyder AZ, Powers WJ, Gusnard DA, Shulman GL (2001). A default mode of brain function. Proc Natl Acad Sci USA.

[62] Craig AD (2003). Interoception: the sense of the physiological condition of the body. Curr Opin Neurobiol.

[63] Craig AD (2009). How do you feel–now? The anterior insula and human awareness. Nat Rev Neurosci.

[64] Seeley WW, Menon V, Schatzberg AF, Keller J, Glover GH, Kenna H (2007). Dissociable intrinsic connectivity networks for salience processing and executive control. J Neurosci.

[65] Turner GR, Spreng RN (2015). Prefrontal engagement and reduced default network suppression co-occur and are dynamically coupled in older adults: the default-executive coupling hypothesis of aging. J Cogn Neurosci.

[66] Menon V, Uddin LQ (2010). Saliency, switching, attention and control: a network model of insula function. Brain Struct Funct.

[67] Bauer CCC, Rozenkrantz L, Caballero C, Nieto‐Castanon A, Scherer E, West MR (2020). Mindfulness training preserves sustained attention and resting state anticorrelation between default‐mode network and dorsolateral prefrontal cortex: a randomized controlled trial. Hum Brain Mapp.

[68] Doll A, Hölzel BK, Boucard CC, Wohlschläger AM, Sorg C (2015). Mindfulness is associated with intrinsic functional connectivity between default mode and salience networks. Front Hum Neurosci.

[69] Parkinson TD, Kornelsen J, Smith SD (2019). Trait mindfulness and functional connectivity in cognitive and attentional resting state networks. Front Hum Neurosci.

[70] Shaurya Prakash R, De Leon AA, Klatt M, Malarkey W, Patterson B (2013). Mindfulness disposition and default-mode network connectivity in older adults. Soc Cogn Affect Neurosci.

[71] Creswell JD, Way BM, Eisenberger NI, Lieberman MD (2007). Neural correlates of dispositional mindfulness during affect labeling. Psychosom Med.

[72] Dickenson J, Berkman ET, Arch J, Lieberman MD (2013). Neural correlates of focused attention during a brief mindfulness induction. Soc Cogn Affect Neurosci.

[73] Pagnoni G (2012). Dynamical properties of BOLD activity from the ventral posteromedial cortex associated with meditation and attentional skills. J Neurosci.

[74] Manna A, Raffone A, Perrucci MG, Nardo D, Ferretti A, Tartaro A (2010). Neural correlates of focused attention and cognitive monitoring in meditation. Brain Res Bull.

[75] Weder BJ (2022). Mindfulness in the focus of the neurosciences - the contribution of neuroimaging to the understanding of mindfulness. Front Behav Neurosci.

[76] Holzel BK, Ott U, Hempel H, Hackl A, Wolf K, Stark R (2007). Differential engagement of anterior cingulate and adjacent medial frontal cortex in adept meditators and non-meditators. Neurosci Lett.

[77] Bolt T, Nomi JS, Rubinov M, Uddin LQ (2017). Correspondence between evoked and intrinsic functional brain network configurations. Hum Brain Mapp.

[78] Cohen JR, D’Esposito M (2016). The segregation and integration of distinct brain networks and their relationship to cognition. J Neurosci.

[79] Di X, Gohel S, Kim EH, Biswal BB (2013). Task vs. rest-different network configurations between the coactivation and the resting-state brain networks. Front Hum Neurosci.

[80] Hearne LJ, Cocchi L, Zalesky A, Mattingley JB (2017). Reconfiguration of brain network architectures between resting-state and complexity-dependent cognitive reasoning. J Neurosci.

[81] Kannurpatti SS, Rypma B, Biswal BB (2012). Prediction of task-related BOLD fMRI with amplitude signatures of resting-state fMRI. Front Syst Neurosci.

[82] Mennes M, Kelly C, Zuo XN, Di Martino A, Biswal BB, Castellanos FX (2010). Inter-individual differences in resting-state functional connectivity predict task-induced BOLD activity. Neuroimage.

[83] Tavor I, Parker Jones O, Mars RB, Smith SM, Behrens TE, Jbabdi S (2016). Task-free MRI predicts individual differences in brain activity during task performance. Science.

[84] Ito T, Kulkarni KR, Schultz DH, Mill RD, Chen RH, Solomyak LI (2017). Cognitive task information is transferred between brain regions via resting-state network topology. Nat Commun.

[85] Harrewijn A, Abend R, Linke J, Brotman MA, Fox NA, Leibenluft E (2020). Combining fMRI during resting state and an attention bias task in children. Neuroimage.

[86] Cheng HJ, Ng KK, Qian X, Ji F, Lu ZK, Teo WP (2021). Task-related brain functional network reconfigurations relate to motor recovery in chronic subcortical stroke. Sci Rep.

[87] Schultz DH, Cole MW (2016). Higher intelligence is associated with less task-related brain network reconfiguration. J Neurosci.

[88] Zuo N, Yang Z, Liu Y, Li J, Jiang T (2018). Core networks and their reconfiguration patterns across cognitive loads. Hum Brain Mapp.

[89] Kiken LG, Garland EL, Bluth K, Palsson OS, Gaylord SA (2015). From a state to a trait: Trajectories of state mindfulness in meditation during intervention predict changes in trait mindfulness. Personal Individ Differences.

[90] Hiroyasu T, Hiwa S. Brain functional state analysis of mindfulness using graph theory and functional connectivity. In 2017 AAAI Spring Symposium Series. 2017;75–680.

[91] Farb NA, Segal ZV, Mayberg H, Bean J, McKeon D, Fatima Z (2007). Attending to the present: mindfulness meditation reveals distinct neural modes of self-reference. Soc Cogn Affect Neurosci.

[92] Kozasa EH, Sato JR, Lacerda SS, Barreiros MA, Radvany J, Russell TA (2012). Meditation training increases brain efficiency in an attention task. Neuroimage.

[93] Tang YY, Holzel BK, Posner MI (2015). The neuroscience of mindfulness meditation. Nat Rev Neurosci.

[94] Wheeler MS, Arnkoff DB, Glass CR (2017). The neuroscience of mindfulness: how mindfulness alters the brain and facilitates emotion regulation. Mindfulness.

[95] Young KS, van der Velden AM, Craske MG, Pallesen KJ, Fjorback L, Roepstorff A (2018). The impact of mindfulness-based interventions on brain activity: a systematic review of functional magnetic resonance imaging studies. Neurosci Biobehav Rev.

[96] Nasreddine ZS, Phillips NA, Bédirian V, Charbonneau S, Whitehead V, Collin I (2005). The Montreal Cognitive Assessment, MoCA: a brief screening tool for mild cognitive impairment. J Am Geriatrics Soc.

[97] Folstein MF, Folstein SE, McHugh PR (1975). Mini-mental state”: a practical method for grading the cognitive state of patients for the clinician. J Psychiatr Res.

[98] Buysse DJ, Reynolds CF, Monk TH, Berman SR, Kupfer DJ (1989). The Pittsburgh Sleep Quality Index: a new instrument for psychiatric practice and research. Psychiatry Res.

[99] Ong JC. Mindfulness-based therapy for insomnia. American Psychological Association; 2017.

[100] Baer RA, Smith GT, Hopkins J, Krietemeyer J, Toney L (2006). Using self-report assessment methods to explore facets of mindfulness. Assessment.

[101] Levinson DB, Stoll EL, Kindy SD, Merry HL, Davidson RJ. A mind you can count on: validating breath counting as a behavioral measure of mindfulness. Front Psychol. 2014;5:1202. 10.3389/fpsyg.2014.01202 PMC4208398 25386148

[102] Wong KF, Massar SA, Chee MW, Lim J (2018). Towards an objective measure of mindfulness: replicating and extending the features of the breath-counting task. Mindfulness.

[103] Lim J, Doshi K. Breath Counting Task (BCT). In Medvedev ON, et al., editors. Handbook of assessment in mindfulness research. Cham: Springer International Publishing; 2022. pp 1–13.

[104] Wong KF, Massar SAA, Chee MWL, Lim J (2018). Towards an objective measure of mindfulness: replicating and extending the features of the breath-counting task. Mindfulness.

[105] Fischl B (2012). FreeSurfer. NeuroImage.

[106] Jenkinson M, Beckmann CF, Behrens TE, Woolrich MW, Smith SM (2012). Fsl. Neuroimage.

[107] Smith SM, Jenkinson M, Woolrich MW, Beckmann CF, Behrens TE, Johansen-Berg H (2004). Advances in functional and structural MR image analysis and implementation as FSL. Neuroimage.

[108] Kong R, Li J, Orban C, Sabuncu MR, Liu H, Schaefer A (2019). Spatial topography of individual-specific cortical networks predicts human cognition, personality, and emotion. Cereb Cortex.

[109] Power JD, Barnes KA, Snyder AZ, Schlaggar BL, Petersen SE (2012). Spurious but systematic correlations in functional connectivity MRI networks arise from subject motion. NeuroImage.

[110] Power JD, Mitra A, Laumann TO, Snyder AZ, Schlaggar BL, Petersen SE (2014). Methods to detect, characterize, and remove motion artifact in resting state fMRI. Neuroimage.

[111] Power JD, Schlaggar BL, Petersen SE (2015). Recent progress and outstanding issues in motion correction in resting state fMRI. Neuroimage.

[112] Van Dijk KRA, Hedden T, Venkataraman A, Evans KC, Lazar SW, Buckner RL (2010). Intrinsic functional connectivity as a tool for human connectomics: theory, properties, and optimization. J Neurophysiol.

[113] Cole MW, Bassett DS, Power JD, Braver TS, Petersen SE (2014). Intrinsic and task-evoked network architectures of the human brain. Neuron.

[114] Schaefer A, Kong R, Gordon EM, Laumann TO, Zuo XN, Holmes AJ (2018). Local-global parcellation of the human cerebral cortex from intrinsic functional connectivity MRI. Cereb Cortex.

[115] Tzourio-Mazoyer N, Landeau B, Papathanassiou D, Crivello F, Etard O, Delcroix N (2002). Automated anatomical labeling of activations in SPM using a macroscopic anatomical parcellation of the MNI MRI single-subject brain. Neuroimage.

[116] Choi EY, Yeo BT, Buckner RL (2012). The organization of the human striatum estimated by intrinsic functional connectivity. J Neurophysiol.

[117] Bastien CH, Vallières A, Morin CM (2001). Validation of the Insomnia Severity Index as an outcome measure for insomnia research. Sleep Med.

[118] Visted E, Vøllestad J, Nielsen MB, Nielsen GH (2014). The impact of group-based mindfulness training on self-reported mindfulness: a systematic review and meta-analysis. Mindfulness.

[119] Jang JH, Jung WH, Kang DH, Byun MS, Kwon SJ, Choi CH (2011). Increased default mode network connectivity associated with meditation. Neurosci Lett.

[120] Wells RE, Yeh GY, Kerr CE, Wolkin J, Davis RB, Tan Y (2013). Meditation’s impact on default mode network and hippocampus in mild cognitive impairment: a pilot study. Neurosci Lett.

[121] Berkovich-Ohana A, Harel M, Hahamy A, Arieli A, Malach R (2016). Alterations in task-induced activity and resting-state fluctuations in visual and DMN areas revealed in long-term meditators. Neuroimage.

[122] Soltani A, Murray JD, Seo H, Lee D (2021). Timescales of cognition in the brain. Curr Opin Behav Sci.

[123] Goldberg SB, Wielgosz J, Dahl C, Schuyler B, MacCoon DS, Rosenkranz M (2016). Does the Five Facet Mindfulness Questionnaire measure what we think it does? Construct validity evidence from an active controlled randomized clinical trial. Psychological Assess.

[124] Isbel B, Stefanidis K, Summers MJ (2020). Assessing mindfulness: experimental support for the discriminant validity of breath counting as a measure of mindfulness but not self-report questionnaires. Psychological Assess.

[125] Grossman P (2008). On measuring mindfulness in psychosomatic and psychological research. J Psychosom Res.

[126] Grossman P, Van Dam NT. Mindfulness, by any other name…: trials and tribulations of sati in western psychology and science. In Mindfulness. Routledge; 2013. pp 219–39.

[127] Van Dam NT, Earleywine M, Danoff-Burg S (2009). Differential item function across meditators and non-meditators on the Five Facet Mindfulness Questionnaire. Personal Individ Differences.

[128] Bergomi C, Tschacher W, Kupper Z (2013). The assessment of mindfulness with self-report measures: existing scales and open issues. Mindfulness.

[129] Tomasino B, Fregona S, Skrap M, Fabbro F (2012). Meditation-related activations are modulated by the practices needed to obtain it and by the expertise: an ALE meta-analysis study. Front Hum Neurosci.

[130] Mooneyham BW, Mrazek MD, Mrazek AJ, Schooler JW (2016). Signal or noise: brain network interactions underlying the experience and training of mindfulness. Ann NY Acad Sci.

[131] Bremer B, Wu Q, Mora Alvarez MG, Holzel BK, Wilhelm M, Hell E (2022). Mindfulness meditation increases default mode, salience, and central executive network connectivity. Sci Rep.

[132] Lim J, Teng J, Patanaik A, Tandi J, Massar SAA (2018). Dynamic functional connectivity markers of objective trait mindfulness. Neuroimage.

[133] Marusak HA, Elrahal F, Peters CA, Kundu P, Lombardo MV, Calhoun VD (2018). Mindfulness and dynamic functional neural connectivity in children and adolescents. Behav Brain Res.

