# Supplementary material for: Mindfulness-based therapy improves brain functional network reconfiguration efficiency
Source: Transl Psychiatry. 2023 Nov 11;13:345. doi: 10.1038/s41398-023-02642-9 (PMC10640625; doi:10.1038/s41398-023-02642-9)
Supplement: Supplementary file 1 — Supplementary materials [file 41398_2023_2642_MOESM1_ESM.docx]

**Supplementary Table 1. Time and interaction effects for all behavioral measures.**

| **Behavioral measure** | ***β*_Timepoint_** | ***p*_perm_** | ***β*_Group x Timepoint_** | ***p*_perm_** |
| --- | --- | --- | --- | --- |
| BCT accuracy (in scanner) | -0.03 | 0.147 | -0.01 | 0.404 |
| BCT accuracy (outside scanner) | 0.06 | 0.070 | -0.02 | 0.340 |
| **FFMQ Total** | **6.44** | **<0.001** | -2.79 | 0.201 |
| **FFMQ Observing** | **2.32** | **0.001** | **-2.23** | **0.032** |
| FFMQ Describing | 0.76 | 0.121 | -0.67 | 0.231 |
| **FFMQ Awareness** | **1.32** | **0.044** | -0.10 | 0.448 |
| FFMQ NonJudging | 0.96 | 0.151 | 1.00 | 0.206 |
| FFMQ NonReacting | 1.08 | 0.081 | -0.78 | 0.262 |
| **PSQI** | **-3.88** | **<0.001** | 1.05 | 0.259 |
| **ISI** | **-6.00** | **<0.001** | **4.22** | **0.006** |

Time and interaction effects from linear mixed models for mindfulness measures, controlled for age and gender. Significant effects (*p* < 0.05 from 1000 permutations) are shown in bold. *BCT*: Breath counting task, *FFMQ*: Five Facet Mindfulness Questionnaire, *PSQI*: Pittsburgh Sleep Quality Index, *ISI*: Insomnia Symptoms Index.

**Supplementary Table 2. Time and interaction effects for FC similarity of all networks.**

| **Network type** | **Network** | ***β*_Timepoint_** | ***p*_perm_** | ***β*_Group x Timepoint_** | ***p*_perm_** |
| --- | --- | --- | --- | --- | --- |
| Intranetwork | **Executive control** | 0.05 | 0.091 | **-0.10** | **0.032** |
|  | **Default mode** | 0.07 | 0.055 | **-0.13** | **0.013** |
|  | Dorsal attention | -0.02 | 0.314 | -0.01 | 0.427 |
|  | Limbic | 0.04 | 0.256 | -0.07 | 0.236 |
|  | **Salience** | 0.06 | 0.054 | **-0.11** | **0.027** |
|  | Somatomotor | 0.03 | 0.304 | -0.01 | 0.424 |
|  | Temporoparietal | 0.01 | 0.441 | -0.01 | 0.474 |
|  | Visual | 0.04 | 0.260 | -0.04 | 0.289 |
|  | Subcortical | 0.03 | 0.291 | -0.03 | 0.351 |
| Internetwork | Executive control | 0.02 | 0.240 | -0.07 | 0.079 |
|  | Default mode | 0.03 | 0.179 | -0.06 | 0.087 |
|  | Dorsal attention | -0.01 | 0.435 | -0.03 | 0.287 |
|  | Limbic | 0.01 | 0.359 | -0.01 | 0.467 |
|  | Salience | 0.03 | 0.188 | -0.06 | 0.123 |
|  | Somatomotor | 0.07 | 0.072 | -0.06 | 0.185 |
|  | Temporoparietal | 0.03 | 0.224 | -0.05 | 0.182 |
|  | Visual | 0.02 | 0.354 | -0.02 | 0.369 |
|  | Subcortical | 0.04 | 0.194 | -0.03 | 0.343 |

Time and interaction effects from linear mixed models for functional connectivity (FC) similarity measures, controlled for age and gender. Significant effects (*p* < 0.05 from 1000 permutations) are shown in bold.

Supplementary Table 3. Interaction effects for rest and task FC of all networks.

| **Network type** | **Network** | **Rest** | | **Task** | |
| --- | --- | --- | --- | --- | --- |
|  |  | **Estimate** | ***p* value** | **Estimate** | ***p* value** |
| Intranetwork | Executive control | 0.00 | 0.457 | 0.00 | 0.503 |
|  | Default mode | **-0.04** | **0.012** | 0.01 | 0.393 |
|  | Dorsal attention | 0.01 | 0.287 | 0.03 | 0.102 |
|  | Limbic | 0.01 | 0.390 | 0.01 | 0.458 |
|  | Salience | -0.01 | 0.339 | 0.00 | 0.420 |
|  | Somatomotor | -0.06 | 0.063 | -0.01 | 0.381 |
|  | Temporoparietal | -0.06 | 0.070 | -0.01 | 0.427 |
|  | Visual | 0.02 | 0.245 | -0.03 | 0.244 |
|  | Subcortical | 0.00 | 0.411 | 0.00 | 0.495 |
| Internetwork | Executive control | 0.00 | 0.257 | 0.01 | 0.241 |
|  | Default mode | 0.00 | 0.445 | -0.01 | 0.206 |
|  | Dorsal attention | 0.00 | 0.364 | 0.01 | 0.075 |
|  | Limbic | -0.01 | 0.074 | -0.01 | 0.202 |
|  | Salience | 0.00 | 0.497 | 0.01 | 0.189 |
|  | Somatomotor | 0.00 | 0.481 | **0.01** | **0.048** |
|  | Temporoparietal | -0.01 | 0.132 | 0.01 | 0.236 |
|  | Visual | 0.01 | 0.223 | 0.01 | 0.060 |
|  | Subcortical | 0.00 | 0.447 | 0.00 | 0.487 |

Group-time interaction effects from linear mixed models for rest and task FC averaged over ROIs in each network, controlled for age and gender. Significant effects (p < 0.05 from 1000 permutations) are shown in bold.

**Supplementary Table 4. Association between mindfulness measures and FC similarity.**

| **Model type** | **Behavioral measure** | **Network** | ***β*_FC sim_** | ***p*_perm_** | ***β*_Group x FC sim_** | ***p*_perm_** |
| --- | --- | --- | --- | --- | --- | --- |
| No group term | FFMQ Total | Executive control | -7.84 | 0.604 | - | - |
|  |  | Default mode | -2.51 | 0.441 | - | - |
|  |  | Salience | -2.82 | 0.547 | - | - |
|  | FFMQ Awareness | Executive control | 1.65 | 0.300 | - | - |
|  |  | Default mode | 1.25 | 0.474 | - | - |
|  |  | Salience | 1.82 | 0.505 | - | - |
|  | FFMQ Observing | Executive control | -0.81 | 0.325 | - | - |
|  |  | Default mode | 1.80 | 0.720 | - | - |
|  |  | Salience | -2.41 | 0.144 | - | - |
| With group term | FFMQ Total | Executive control | -1.93 | 0.733 | -15.44 | 0.232 |
|  |  | Default mode | 2.74 | 0.311 | -18.17 | 0.201 |
|  |  | Salience | -1.64 | 0.564 | -6.35 | 0.368 |
|  | FFMQ Awareness | Executive control | 2.49 | 0.384 | -1.49 | 0.418 |
|  |  | Default mode | 1.95 | 0.428 | -1.55 | 0.432 |
|  |  | Salience | 2.91 | 0.355 | -1.80 | 0.398 |
|  | FFMQ Observing | Executive control | -0.02 | 0.553 | -4.33 | 0.296 |
|  |  | Default mode | 1.70 | 0.541 | -3.55 | 0.348 |
|  |  | Salience | -8.06 | 0.013 | 6.36 | 0.152 |

Coefficients for FC similarity and interaction term (Group x FC similarity) from linear models testing for association of changes of mindfulness measures with changes in FC similarity after intervention, controlled for age and gender. Only measures showing significant time and/or interaction effects were included in this analysis (i.e., FFMQ total score, FFMQ Awareness subscale score, FFMQ Observing subscale score, executive control network, default mode network and salience network intranetwork FC similarity). No significant positive associations (*p* < 0.05 from 1000 permutations) were found, even when accounting for possible interaction effects across intervention groups. *FFMQ*: Five Facet Mindfulness Questionnaire.

**Supplementary Table 5. Association of change in FC similarity with change in sleep indices.**

| **Model type** | **Behavioral measure** | **Network** | ***β*_FC sim_** | ***p*_perm_** | ***β*_Group x FC sim_** | ***p*_perm_** |
| --- | --- | --- | --- | --- | --- | --- |
| No group term | PSQI | Executive control | -4.55 | 0.129 | - | - |
|  |  | Default mode | -4.02 | 0.352 | - | - |
|  |  | Salience | -4.45 | 0.078 | - | - |
|  | ISI | Executive control | -1.33 | 0.434 | - | - |
|  |  | Default mode | -2.82 | 0.690 | - | - |
|  |  | Salience | -2.25 | 0.644 | - | - |
| With group term | PSQI | Executive control | -3.56 | 0.49 | -0.74 | 0.489 |
|  |  | Default mode | -3.08 | 0.565 | -0.68 | 0.498 |
|  |  | Salience | -2.30 | 0.609 | -2.59 | 0.362 |
|  | ISI | Executive control | -1.30 | 0.487 | 5.28 | 0.288 |
|  |  | Default mode | -1.32 | 0.638 | 4.16 | 0.338 |
|  |  | Salience | 0.18 | 0.290 | 1.24 | 0.415 |

Coefficients for FC similarity and interaction term (Group x FC similarity) from linear models testing for association of changes of sleep measures with changes in FC similarity after intervention, controlled for age and gender. Only rest-task reconfiguration measures showing significant time and/or interaction effects were included in this analysis (i.e., executive control, default mode and salience network intranetwork FC similarity). No significant positive associations (*p* < 0.05 from 1000 permutations) were found, even when accounting for possible interaction effects across intervention groups. *PSQI*: Pittsburgh Sleep Quality Index, *ISI*: Insomnia Symptoms Index.
